# Quantitative Assessment of Choroidal Parameters in Patients with Various Types of Diabetic Macular Oedema: A Single-Centre Cross-Sectional Analysis

**DOI:** 10.3390/biology10080725

**Published:** 2021-07-29

**Authors:** Diana Anna Dmuchowska, Patryk Sidorczuk, Barbara Pieklarz, Joanna Konopińska, Zofia Mariak, Iwona Obuchowska

**Affiliations:** Ophthalmology Department, Medical University of Bialystok, 24a M. Sklodowskiej-Curie, 15-276 Bialystok, Poland; barbara.pieklarz@gmail.com (B.P.); joannakonopinska@o2.pl (J.K.); mariakzo@umb.edu.pl (Z.M.); iwonaobu@wp.pl (I.O.)

**Keywords:** choroid, choroidal thickness, choroidal volume, choroidal vascularity index, diabetic macular oedema, cystoid macular oedema, diffuse macular oedema, subretinal fluid, OCT

## Abstract

**Simple Summary:**

Choroidopathy is one of the components in the pathogenesis of diabetic macular oedema (DME). This study investigated the optical coherence tomography-based choroidal parameters: thickness, volume, choroidal vascularity index (CVI), luminal area (LA), stromal area (SA), and total choroidal area (TCA) in relation to the presence and type of DME (cystoid, diffuse, and with subretinal fluid). Diabetic choroidopathy seems to play a role in the development of DME but is less likely involved in the pathogenesis of specific types thereof.

**Abstract:**

Diabetic macular oedema (DME) is an outcome of multiple, complex and not fully understood mechanisms. The aim of this study was to define the role of choroidopathy in the pathogenesis of various DME types. The retrospective cross-sectional single-centre study included 140 eyes from 105 patients with DME and 76 eyes from 52 non-diabetic controls. The eyes were stratified according to the type of DME: cystoid, diffuse, and with subretinal fluid. Optical coherence tomography-based choroidal parameters: thickness, volume, choroidal vascularity index (CVI), luminal area (LA), stromal area (SA), and total choroidal area (TCA) were compared. Eyes with DME, regardless of the type thereof, had lower choroidal thickness, volume, and CVI values than the controls. Further, the eyes with some specific DME types differed significantly from the controls in terms of LA and SA. While the eyes with various DME types did not differ significantly in terms of their choroidal thickness, volume and CVI, some between-group differences were found in LA, SA and TCA. Diabetic choroidopathy seems to play a role in the development of DME but is less likely involved in the pathogenesis of specific types thereof.

## 1. Introduction

The retina is supplied with blood from two independent vascular beds. The inner retina is supplied by the central retinal artery. Choriocapillaris circulation provides the blood for the high-energy demanding outer retina, a region of utmost importance for visual acuity [1]. Diabetes mellitus affects negatively both vascular systems mentioned above [2,3]. Analogically to retinal abnormalities, diabetes may result in choroidal changes, such as microaneurysms, dilatation, and obstruction of the vessels, vascular remodelling with increased vascular tortuosity, vascular dropout, focal vascular non-perfusion, and choroidal vascularisation [4,5,6]. The histological findings are consistent with abnormalities observed on optical coherence tomography (OCT) [7,8]. Choroidopathy may trigger the development of retinopathy due to retinal tissue hypoxia and overexpression of vascular endothelial growth factor (VEGF); this contributes to further retinal damage and diabetic macular oedema (DME) [5]. However, the exact relationship between the choroidal abnormalities and the presence of DME, and especially the type thereof, remains unclear.

DME can occur at any stage of diabetic retinopathy (DR). Based on OCT findings, DME can be classified into three types: sponge-like retinal swelling, cystoid macular oedema, and serous retinal detachment [9]. Macular oedema is defined as an abnormal increase in the volume of intra- and extracellular fluid in the macula. It results from an imbalance between the fluid entry and exit, as well as from retinal hydraulic conductivity. Retinal homeostasis is necessary for tissue transparency and light transmission. DME is caused primarily by the breakdown of the blood–retinal barrier [10]. The inner blood–retinal barrier plays a pivotal role in the control of fluid entry into the retina. The barrier is formed by tight junctions between the endothelial cells of retinal vessels and regulated by a neuro-glio-vascular complex composed of pericytes, retinal glial Müller cells, astrocytes, and microglia [11,12]. The outer blood–retinal barrier is the intercellular junction complex of the retinal pigment epithelium (RPE) accompanied by the external limiting membrane (ELM) [13]. The breakdown of the inner and outer blood–retinal barrier may occur simultaneously, under the influence of various factors, receptors, and signalling pathways [10]. These molecular and cellular mechanisms affect not only the retinal and choroidal vessels and RPE but also neurons and glial cells [10]. Thus, DME may be triggered by three mechanisms: ischaemia, neurodegeneration, and oedema [14].

Available data on choroidal thickness in the course of diabetic retinopathy are contradictory, and further affected by the presence of DME [15,16,17,18,19,20,21,22,23,24,25,26,27]. Moreover, it should be remembered that choroidal thickness is a rough estimate rather than an accurate marker of choroidal status. Therefore, in this study, we not only determined the choroidal thickness and volume but also calculated the choroidal vascularity index (CVI). CVI is a novel OCT-based choroidal quantitative parameter providing more detailed information about the vascular constituent of the choroid across all its layers: choriocapillaris, Sattler’s layer, and Haller’s layer. CVI is defined as the ratio of the luminal area (LA) to the total choroidal area (TCA) [28,29]. It has been proposed as a marker for early diagnosis, progression monitoring, and stratification of patients with various retinal and choroidal diseases and systemic conditions, including those of vascular or inflammatory origin [28]. Choroidal thickness and consequently volume depend on various physiological and pathological factors, including age, ethnicity, sex, refraction, axial length, time of day, DME, DR severity, panretinal photocoagulation (PRP), and systolic blood pressure [28,29,30,31,32]. On the contrary, CVI is modified only by subfoveal choroidal thickness and may depend on the patient’s age. Hence, CVI can be considered a relatively stable parameter to evaluate changes in the choroidal vasculature [33]. Unlike the relationship between choroidal thickness and DME, which has been the subject of many previous studies [15,16,17,18,19,20,21,22,23,24,25,26,27], only a few authors have analysed choroidal thickness depending on DME type [27,34,35] and even fewer have studied CVI in various DME types [34,35].

The aim of this study was to define the role of choroidopathy in the pathogenesis of various DME types. The OCT-based choroidal characteristics (choroidal thickness, choroidal volume, and other parameters) were analysed in relation to the presence of DME and type thereof. A better insight into the complex pathogenesis of DME may facilitate patient stratification in terms of the risk of occurrence, progression, or treatment response. Further, in the era of personalised medicine, each of the disease pathways should optimally be quantifiable to enable early and individualised treatment [36]. We believe that the results of this study might constitute a basis for novel treatment modalities.

## 2. Materials and Methods

### 2.1. Study Design

This retrospective single-centre cross-sectional study included 140 eyes from 105 patients with type 1 and 2 diabetes mellitus who underwent same-day fluorescein angiography and OCT at the Department of Ophthalmology, University Teaching Hospital of Bialystok (Poland) between 28 February 2017 and 20 February 2021. The patients’ eyes were stratified into three groups according to the DME type: (1) diffuse DME, (2) cystoid DME, and (3) DME with subretinal fluid [9]. The age-, sex- and spherical equivalent-matched control group comprised 76 eyes from 52 non-diabetic patients undergoing routine ophthalmological assessments.

The exclusion criteria included: tractional DME, a history of prior posterior segment surgery or intravitreal injections, macular laser photocoagulation, panretinal photocoagulation less than 3 months prior to the examination, ametropia ≥ 3.0 dioptres, other ocular diseases affecting macula, glaucoma, known ocular or systemic pathology potentially able to modulate choroidal vasculature, and insufficient quality of fluorescein angiograms or OCT images. The study protocol was previously described in detail by Sidorczuk et al. [37].

The protocol of the study was reviewed and approved by the Local Bioethics Committee at the Medical University of Bialystok (decision no. APK.002.216.2020) and conformed with the provisions of the Declaration of Helsinki. On the day of the examination, written informed consent was provided by all patients involved in the study.

### 2.2. OCT Images Acquisition and Analysis

OCT images were taken in mydriasis between 8 a.m. and 11 a.m. to avoid diurnal variation in the choroidal thickness and volume. The images were independently assessed by two investigators (D.A.D. and P.S.) blinded to the clinical characteristics of examined eyes.

**Figure 1:**
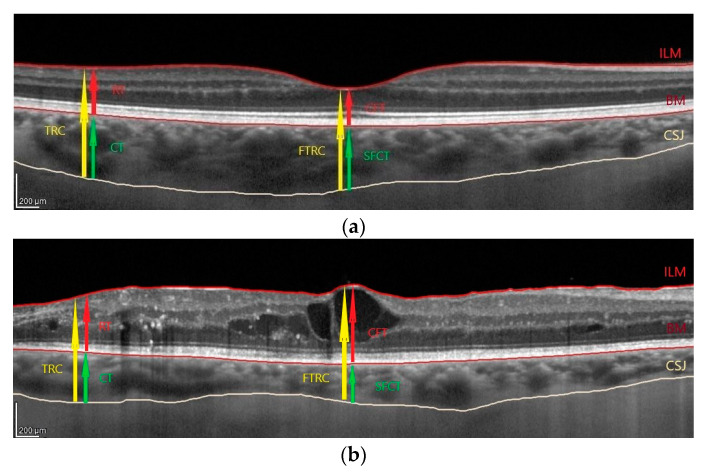

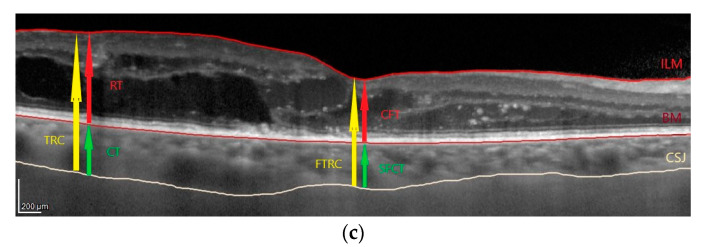
Representative OCT scans across the fovea representing measurements and types of diabetic macular oedema (DME): (**a**) control; (**b**) cystoid; (**c**) diffuse. DME with subretinal fluid was classified as such regardless of concomitant cystoid or diffuse oedema. In cases of mixed cystoid and diffuse oedema, the eye was classified according to the predominant type. As described previously [37], internal limiting membrane (ILM) and Bruch’s membrane (BM) were detected automatically, while the choroidal-scleral junction (CSJ) was marked manually. Subfoveal choroidal thickness (SFCT) was defined as the distance between the BM and the CSJ at the fovea (green arrow). The SFCT was manually measured using the calliper tool and calculated as the difference between the foveal total thickness (FTRC, retinal + choroidal thickness, from the ILM to the CSJ; yellow arrow) and the central foveal thickness (CFT; red arrow). Analogically, the choroidal thickness outside the fovea (CT; green arrow) was calculated by subtracting the retinal thickness (RT; red arrow) from the total thickness (TRC, retinal + choroidal thickness, from the ILM to the CSJ, yellow arrow).

Spectral domain-OCT was carried out with a Spectralis HRA + OCT imaging device (Heidelberg Engineering, Heidelberg, Germany). As described in our previous publication [37], the protocol of OCT imaging comprised of 25 horizontal raster scans (20 × 20°) and a linear B-scan centred at the fovea, as shown in Figure 1.The values of choroidal parameters were obtained by subtracting retinal parameters from the sum of retinal and choroidal parameters. Averaged thickness and volume maps were created automatically according to the conventional Early Treatment Diabetic Retinopathy Study Research Group (ETDRS) grid with nine subfields [38]. The fovea was checked and replotted manually whenever necessary.

Three types of DME were identified based on OCT findings (Figure 1), according to Otani et al. A sponge-like retinal swelling (diffuse oedema) was determined as the increased retinal thickness with reduced intraretinal reflectivity and expanded areas of lower reflectivity that were prominent in the outer retinal layers. Cystoid macular oedema was characterised by the presence of intraretinal cystoid spaces at the macular area that cause the fovea to protrude. Serous retinal detachment was seen as subretinal fluid accumulation with a distinct outer border of the detached retina [9]. Whenever an OCT image satisfied the criteria of both diffuse and cystoid oedema, the eye was classified according to the predominant type. Eyes with subretinal fluid were classified as such regardless of concomitant cystoid or diffuse macular oedema.

The major part of the choroid are vessels organised in three vascular layers. The choriocapillaris, a thin capillary network, is the innermost layer, the medium- and small-sized vessels form the intermediate Sattler’s layer, and the large vessels form the outermost Haller’s layer. The suprachoroid separates the latter layer from the sclera [10]. CVI reflects the vascularity of the choroid [28,39]. To calculate CVI, choroidal areas on OCT scans were binarised with a modified Niblack method, as described by Sonoda et al. [29,40]. Briefly, the entire horizontal scan (6 mm) across the fovea was assessed, with the BM as the upper margin and the choroidal-scleral junction as the lower margin. Binarisation and segmentation of the images were done with ImageJ software (http://imagej.nih.gov/ij (accessed on 6 April 2020), version 1.49). LA, stromal area (SA), and TCA were calculated, and the CVI was determined as the LA to TCA ratio.

The inter-grader reliability was measured by the absolute agreement model of the intraclass correlation coefficient (ICC). ICC value for choroidal thickness, volume, and CVI were greater than 0.8, which indicated good agreement. Since CVI is calculated from LA, SA, and TCA, ICC was not computed for the latter three parameters. With Bland–Altman plot analyses, the fixed and proportional biases were excluded.

### 2.3. Fluorescein Angiograms Acquisition and Analysis

Fluorescein angiography was performed with a Spectralis HRA + OCT imaging device (Heidelberg Engineering, Heidelberg, Germany) according to the standard procedure. The images were used to assess the severity of DR according to the ETDRS criteria [38,41].

### 2.4. Statistical Analysis

Statistical analyses were performed with R software, version 3.5.1 (http://cran.r-project.org). Descriptive statistics included numbers (% of each group) for nominal variables and means ± standard deviations (SD) for continuous variables. The normality of the distribution was verified with the Shapiro–Wilk test, on the basis of skewness and kurtosis values, as well as based on visual inspection of histograms. Between-group comparisons were performed with the Fisher exact test for nominal variables and ANOVA for continuous variables. Univariate linear mixed-effects models were developed to compare the DME types (cystoid vs. diffuse vs. subretinal fluid vs. controls), with random effects for the correlation of two eyes from the same patient. A range of univariate models with choroidal parameters as independent variables and age, sex, PRP, and DR severity as covariates was created as well. All tests were two-tailed with α = 0.05.

## 3. Results

### 3.1. Baseline Characteristics

Demographic and clinical characteristics of the study patients are presented in Table 1. The study groups were age-, sex-, and spherical equivalent-matched and did not differ significantly in terms of DR severity or PRP distribution.

### 3.2. Choroidal Parameters in Eyes with Various Types of DME and Non-Diabetic Controls

**Figure 2:**
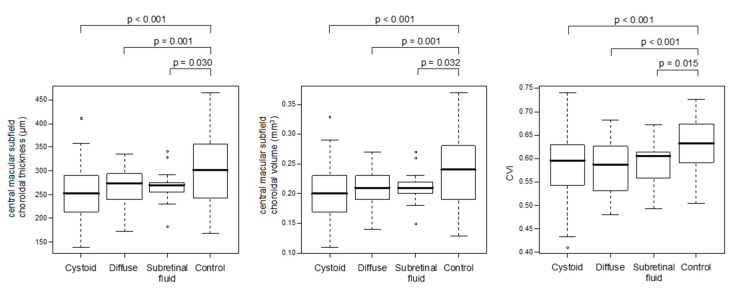
Selected choroidal parameters in eyes with various types of DME and non-diabetic controls. Hollow dots indicate outlier observations (i.e., values exceeding 1.5 the interquartile range above the upper quartile and bellow the lower quartile); *p*-values from the univariate mixed-effect models (with covariates) comparing the study groups identified based on the type of DME with non-diabetic controls; only *p*-values < 0.05 are marked; covariates included in each model: age in years, sex (male/female), DR (NPDR/PDR), PRP (no/yes). CVI = choroidal vascularity index.

Patients with DME differed significantly from controls in terms of choroidal thickness and volume and other choroidal parameters (Table 2 and Figure 2). Compared with controls, patients with DME, regardless of the type thereof, presented with thinner choroids and consequently lower choroidal volumes across all the ETDRS subfields. Moreover, the presence of DME was associated with lower CVI values, either due to a decrease in LA or due to an increase in SA.

Choroidal thickness and volume and other choroidal parameters, stratified according to the type of DME, are presented in Table S1. Univariate mixed-effect models without covariates comparing the study groups identified based on the type of DME (cystoid/diffuse/subretinal fluid) with non-diabetic controls are shown in Table S2.

### 3.3. Choroidal Parameters According to the Type of DME

No significant differences in choroidal thickness, choroidal volume, or CVI were found when the results were stratified according to the DME type (Figure 2 and Tables S3–S5). However, the groups with various DME types differed (albeit not necessarily significantly) in terms of other choroidal parameters: LA, SA, and TCA (Table 3). The highest values of LA and TCA were found in the subretinal fluid group, followed by the diffuse and cystoid groups, respectively.

## 4. Discussion

The pathogenesis of DME is complex and multifactorial. In this study, we analysed one component of DME pathogenesis, namely the OCT-based choroidal characteristics, and their relationship with DME and its type.

Our present study confirmed that the type of DME does not correspond to DR severity. DME can develop at any stage of DR [10]. Some previous studies demonstrated that the presence of both DR and DME was associated with a decrease in choroidal thickness [16,17,20,21,22,23]. Our findings are consistent with these observations, as regardless of the DME type, the choroid across all ETDRS fields was thinner than in the controls. Consequently, DME was also associated with a decrease in choroidal volume. Gerendas et al. also found an evident association between DME and choroidal thinning. Similar to our present study, those researchers also observed choroidal thinning over the entire posterior pole [16]. However, some authors have reported an increase in choroidal thickness during DME [15,25,34,35,42], while others have found no significant association between the choroidal thickness and DME [18,24,26,43].

The discrepancies mentioned above might reflect a confounding effect of multiple systemic and ocular variables on choroidal thickness and volume; the list of such potential confounders includes anti-VEGF and steroid treatment and PRP, to mention but a few [5,28,31]. Importantly, our present study included solely the DME treatment-naïve patients, and the examined groups were age-, sex-, and spherical equivalent-matched. Furthermore, some potential confounding factors, namely age, sex, DR severity, and PRP, were considered covariates in the linear mixed-effects models.

Still, little is known about the association between choroidal parameters and the type of DME [27,34,35]. Our present study did not show significant differences in the choroidal thickness and volume in eyes with various DME types. However, choroid in the eyes with DME with subretinal fluid tended to be thicker than in those with cystoid and diffuse DME. In previous studies, DME with subretinal fluid was also shown to be associated with higher values of choroidal parameters [27,34,42]. However, the group of eyes with DME with subretinal fluid analysed in our present study was relatively small (13 eyes), and thus, the results should be interpreted with caution. Moreover, there are some ambiguities regarding the DME with subretinal fluid. According to Arf et al., serous macular detachment should be considered an accompanying morphological finding rather than a type of DME, as it was also found in the eyes with cystoid and diffuse DME [44]. In turn, Vujosevic et al. suggested that DME with and without subretinal fluid represent two distinct morphologic and functional entities [27]. Furthermore, there are some discrepancies regarding the association between subretinal fluid accumulation and DME duration and severity [27,44]. From the pathogenetic perspective, it is unclear in which proportion the accumulation of subretinal fluid results from its increased entry from the choroid and its insufficient removal by the RPE [45].

Choroidal thickness (and consequently volume) present wide inter-individual variability and are dependent on various confounding factors. In our study, patients with cystoid macular oedema and the controls were more numerous than diffuse and subretinal fluid groups, which could lead to greater variability.

CVI is defined as the ratio of LA to TCA, with the latter consisting of LA and SA. Our present study demonstrated alterations in the vascular and stromal component of the choroid in DME eyes. Our findings are consistent with the results of previous studies in which the CVI in diabetic eyes (even without DME) was lower than in the controls [34,46,47]. In our present study, the LA values in the cystoid DME group were lower than in the controls, whereas the eyes with diffuse DME and DME with subretinal fluid had higher SA values than the control eyes. The CVI values did not differ significantly according to the DME type, which is in line with the results reported by Gupta et al. [34] and Kase et al. [35]. While we found between-group differences in LA, SA, and TCA values for eyes with various DME types, none of them had a significant effect on CVI.

The choroidal volume showed a significant reduction in the majority of sectors of diabetic eyes in comparison to controls, whereas the TCA was not significantly different. We assume that this discrepancy might be due to the fact that TCA cannot be extrapolated on the whole ETDRS grid, as it is measured on a single scan passing through the fovea (in our study measured on a 6 mm scan). Choroid in healthy people has a bowl-shaped contour with a maximum thickness at the subfoveal level. S-shaped appearance, various contour alterations, or irregular contour have been reported in patients with DM (with DR/DME). The normally thickest subfoveal location is replaced extrafoveally in 80% of patients with DR, as reviewed in [32].

Decreased LA, similarly to decreased choroidal thickness and volume, can result from the obstruction of the choriocapillaris, vascular dropout, focal vascular non-perfusion, and reduction of the choroidal blood flow [4,5,6,7,8,48]. Doppler flowmetric studies demonstrated that the choroidal blood flow may decrease at the early stages of diabetic retinopathy and is further reduced in the presence of macular oedema [48]. According to Querques et al., the overall thinning of the choroid in diabetic eyes, with DME or without, may lead to tissue hypoxia. This may be reflected by an increase in VEGF synthesis and RPE dysfunction, with a resultant breakdown of the blood–retinal barrier and the development of DME [20,27]. According to Kase et al., a reason behind the SA increase may be the accumulation of advanced glycation end products (AGEs). Finally, choroidal vascular permeability associated with hypoxia and VEGF overproduction may lead to protein leakage into the choroidal stroma [35].

In our present study, the eyes with DME differed significantly from non-diabetic controls in terms of choroidal parameters, but only slight differences in these parameters were found between the groups with various DME types. Thus, one may speculate that while choroidopathy and outer blood-retinal breakdown play a major role in DME development, these processes have little impact in the pathogenesis of specific types of DME Consequently, DME pathogenesis seems to be related to other mechanisms that may be more involved, such as the inner blood–retinal barrier breakdown, neurodegeneration, ischaemia, etc.

The eligibility criteria for this study constitute its strength. Among the analysed groups there was no difference in age, sex, or spherical equivalent. Only treatment-naïve patients were included. All measurements were taken at the same time of the day to avoid diurnal variations. Moreover, both the choroidal thickness and volume over a 6 mm diameter in the macula were evaluated to characterise the choroid. Singh et al. suggested that the choroidal volume assessment is advantageous due to the irregularities of the border between the choroidal and sclera [32]. To evaluate the choroid in more detail, we assessed its luminal and stromal components and calculated the CVI.

The study also has some potential limitations. Due to its retrospective design, the data on the type and duration of diabetes, systolic blood pressure, fasting blood glucose, and glycated haemoglobin (HbA1C) levels were not available. However, as stated by Agrawal et al., CVI does not correlate with these parameters [28]. Choroidal thickness seems to decrease with the duration of DME [31] but might be independent of HbA1C levels [19]. Furthermore, we assessed a single foveal scan to determine CVI. This approach is consistent with findings that CVI is comparable across all the ETDRS subfields [32]. The CVI variation does not follow similar patterns as seen in choroidal thickness and volume in various locations [49]. However, it is unclear which choroidal layers were the source of changes in LA and SA. In future studies, improvements in CVI assessment and fully automated CVI algorithm integrated into the OCT device may facilitate standardisation of this informative parameter, as pointed out by Agrawal et al. [50]. Additionally, an automated and validated segmentation method dedicated to the segmentation of the choroidal layers would be a useful addition. Furthermore, the prospective longitudinal design of the study could facilitate a better understanding of the pathogenesis of DME.

## 5. Conclusions

Some choroidal parameters seem to be altered depending on the presence of DME and the type thereof. Choroidal thickness and volume and other choroidal parameters differed significantly between the eyes with DME and non-diabetic controls. However, the type of DME appeared to be less associated with the choroidal parameters, with some type-specific differences found solely for LA, SA, and TCA. Thus, while diabetic choroidopathy seems to play a role in the development of DME, it is less likely involved in the pathogenesis of specific types of DME. Other mechanisms may be more involved, such as the inner blood–retinal barrier breakdown, neurodegeneration, or ischemia.

## Figures and Tables

**Table 1:** Baseline characteristics of the study patients according to the type of DME.

	Overall	Type of DME			Controls	*p*
		Cystoid	Diffuse	Subretinal Fluid		
Number of patients	157	65	31	9	52	
Number of eyes	216	89	38	13	76	
Age, years, mean ± SD	58.98 ± 14.01	61.17 ± 10.41	60.28 ± 12.44	52.60 ± 16.46	55.73 ± 17.87	0.191
Sex, female, *n* (%)	82 (52.2)	32 (49.2)	16 (51.6)	6 (66.7)	28 (53.8)	0.790
Spherical equivalent, mean ± SD	0.38 ± 1.16	0.24 ± 1.08	0.82 ± 1.05	−0.06 ± 0.95	0.39 ± 1.28	0.198
DR severity, *n* (%)						
NPDR	87 (40.3)	56 (62.9)	21 (55.3)	10 (76.9)	-	0.379
PDR	53 (24.5)	33 (37.1)	17 (44.7)	3 (23.1)	-	
PRP, *n* (%)						
no	167 (77.3)	59 (66.3)	22 (57.9)	10 (76.9)	-	0.379
yes	49 (22.7)	30 (33.7)	16 (42.1)	3 (23.1)	-	

Between-group comparisons with Fisher exact test for nominal variables and ANOVA for continuous variables. DME = diabetic macular oedema; DR = diabetic retinopathy; NPDR = non-proliferative diabetic retinopathy; PDR = proliferative diabetic retinopathy; PRP = panretinal photocoagulation.

**Table 2:** Univariate mixed-effect models (with covariates) comparing the study groups identified based on the type of DME (cystoid/diffuse/subretinal fluid) with non-diabetic controls.

Characteristic	Cystoid vs. Controls (Baseline)			Diffuse vs. Controls (Baseline)			Subretinal Fluid vs. Controls (Baseline)		
	β	SE	*p*	β	SE	*p*	β	SE	*p*
Choroidal thickness (µm):									
outerT	**−47.36**	**9.75**	**<0.001**	**−35.15**	**11.40**	**0.002**	**−31.47**	**14.92**	**0.036**
innerT	**−40.10**	**10.77**	**<0.001**	**−32.90**	**12.67**	**0.010**	−31.47	16.65	0.060
central macular	**−35.78**	**11.26**	**0.002**	**−31.52**	**13.13**	**0.017**	−33.83	17.18	0.051
innerN	**−40.07**	**12.16**	**0.001**	**−38.44**	**14.27**	**0.008**	**−38.03**	**18.64**	**0.043**
outerN	**−39.04**	**13.40**	**0.004**	**−31.48**	**15.39**	**0.042**	−20.46	19.92	0.305
outerS	**−47.74**	**10.86**	**<0.001**	**−38.08**	**12.60**	**0.003**	−32.32	16.41	0.051
innerS	**−42.85**	**10.95**	**<0.001**	**−36.89**	**12.77**	**0.004**	−26.34	16.70	0.116
innerI	**−32.86**	**11.38**	**0.004**	−21.89	13.21	0.099	−33.07	17.23	0.056
outerI	**−30.02**	**11.48**	**0.009**	−19.81	13.28	0.138	−14.78	17.29	0.394
SFCT	**−33.13**	**11.83**	**0.006**	−25.31	14.00	0.072	−28.05	18.42	0.129
Choroidal volume (mm^3^):									
outerT	**−0.25**	**0.05**	**<0.001**	**−0.19**	**0.06**	**0.002**	**−0.17**	**0.08**	**0.038**
innerT	**−0.07**	**0.02**	**<0.001**	**−0.05**	**0.02**	**0.011**	−0.05	0.03	0.059
central macular	**−0.03**	**0.01**	**0.002**	**−0.03**	**0.01**	**0.015**	−0.03	0.01	0.054
innerN	**−0.05**	**0.02**	**0.009**	**−0.006**	**0.02**	**0.007**	−0.005	0.03	0.074
outerN	**−0.22**	**0.07**	**0.002**	**−0.17**	**0.08**	**0.033**	−0.10	0.10	0.329
outerS	**−0.26**	**0.06**	**<0.001**	**−0.21**	**0.06**	**0.001**	**−0.18**	**0.08**	**0.039**
innerS	**−0.07**	**0.02**	**<0.001**	**−0.06**	**0.02**	**0.001**	−0.04	0.03	0.136
innerI	**−0.05**	**0.02**	**0.004**	−0.03	0.02	0.105	−0.05	0.03	0.056
outerI	**−0.17**	**0.06**	**0.007**	−0.11	0.07	0.105	−0.10	0.09	0.247
total	**−1.16**	**0.30**	**<0.001**	**−0.89**	**0.34**	**0.008**	−0.76	0.43	0.079
Other choroidal parameters:									
CVI	**−0.03**	**0.01**	**0.009**	**−0.04**	**0.01**	**0.003**	**−0.04**	**0.02**	**0.027**
LA (mm^2^)	**−0.17**	**0.07**	**0.017**	−0.10	0.08	0.227	0.04	0.11	0.703
SA (mm^2^)	0.0001	0.04	0.998	**0.11**	**0.05**	**0.034**	**0.17**	**0.07**	**0.011**
TCA (mm^2^)	−0.17	0.10	0.099	0.005	0.12	0.965	0.21	0.16	0.176

β—coefficient from the regression model, SE—standard error, *p* < 0.05 highlighted with bold. *n* = 216 eyes (76 eyes in control group, 13 eyes with subretinal fluid, 89 eyes with cystoid macular oedema, and 38 eyes with diffuse macular oedema). Each row represents one model with a factorial response variable (diffuse, cystoid, subretinal fluid) with the control group as a baseline. Covariates included in each model: age in years, sex (male/female), DR (NPDR/PDR), PRP (no/yes). T = temporal; I = inferior; N = nasal; S = superior; SFCT = subfoveal choroidal thickness; CVI = choroidal vascularity index; LA = luminal area; SA = stromal area; TCA = total choroidal area; conventional ETDRS grid with nine subfields: central macular subfield (central field within a 500 µm radius), four inner subfields (within a 500–1500 µm radius), and four outer subfields (within a 1500–3000 µm radius) [38].

**Table 3:** Univariate mixed-effect models (with covariates) comparing the study groups identified based on the type of DME.

Choroidal Parameters	Cystoid vs. Diffuse			Cystoid vs. Subretinal Fluid			Diffuse vs. Subretinal Fluid		
	β	SE	*p*	β	SE	*p*	β	SE	*p*
CVI	−0.008	0.01	0.456	0.01	0.02	0.502	0.002	0.02	0.920
LA (mm^2^)	0.11	0.06	0.099	**−0.20**	**0.10**	**0.047**	−0.12	0.10	0.228
SA (mm^2^)	**0.12**	**0.04**	**0.008**	**−0.17**	**0.07**	**0.011**	−0.07	0.07	0.352
TCA (mm^2^)	**0.21**	**0.09**	**0.021**	**−0.40**	**0.15**	**0.011**	−0.20	0.15	0.192

β—coefficient from the regression model, SE—standard error, *p* < 0.05 highlighted with bold. *n* = 140 eyes (13 eyes with subretinal fluid, 89 eyes with cystoid macular oedema, and 38 eyes with diffuse macular oedema). Covariates included in each model: age in years, sex (male/female), DR (NPDR/PDR), PRP (no/yes). CVI = choroidal vascularity index; LA = luminal area; SA = stromal area; TCA = total choroidal area.

## Data Availability

The data presented in this study are available on request from the corresponding author. Names and exact data of the participants of the study may not be available because of the privacy policy.

## Supplementary Materials

The following are available online at https://www.mdpi.com/article/10.3390/biology10080725/s1—Table S1: Choroidal parameters in study patients, stratified according to the type of DME, Table S2: Univariate mixed-effect models (without covariates) comparing the study groups identified based on the presence of DME and type thereof (cystoid/diffuse/subretinal fluid vs. controls), Table S3: Univariate mixed-effect models (without covariates) comparing the study groups identified based on the type of DME (cystoid vs. subretinal fluid, diffuse vs. subretinal fluid), Table S4: Univariate mixed-effect models (with covariates) comparing the study groups identified based on the type of DME (cystoid vs. subretinal fluid, diffuse vs. subretinal fluid), Table S5. Univariate mixed-effect models (with covariates and without) comparing the study groups identified based on the type of DME (cystoid vs. diffuse).
