# Supplementary material for: Quantitative Assessment of Choroidal Parameters in Patients with Various Types of Diabetic Macular Oedema: A Single-Centre Cross-Sectional Analysis
Source: Biology (Basel). 2021 Jul 29;10(8):725. doi: 10.3390/biology10080725 (PMC8389323; doi:10.3390/biology10080725)
Supplement: Supplementary file 1 [file biology-10-00725-s001.zip › biology-1312965-supplementary.pdf]

Table S1. Choroidal parameters in study patients, stratified according to the type of DME.

| Characteristic | Cystoid | Diffuse | Subretinal fluid | Controls |
|---|---|---|---|---|
| Choroidal thickness (μm): | | | | |
| outerT | 239.69±48.22 | 257.92±47.26 | 257.85±33.13 | 297.45±68.58 |
| innerT | 251.02±51.96 | 266.53±47.65 | 267.46±31.95 | 304.45±70.53 |
| central macular | 254.83±54.13 | 265.61±43.61 | 269.38±39.82 | 303.64±72.77 |
| innerN | 248.17±60.84 | 257.97±49.15 | 258.31±48.13 | 295.67±75.15 |
| outerN | 220.07±63.83 | 237.24±55.21 | 235.23±59.79 | 268.45±80.88 |
| outerS | 256.71±54.68 | 273.34±41.33 | 275.23±41.89 | 315.68±67.03 |
| innerS | 259.07±52.97 | 270.24±40.86 | 278.08±30.58 | 309.99±70.26 |
| innerI | 251.29±55.16 | 265.84±49.75 | 262.08±41.18 | 299.80±74.84 |
| outerI | 241.17±58.63 | 250.03±49.43 | 268.15±62.27 | 289.83±77.48 |
| SFCT | 252.97±57.11 | 268.53±50.81 | 272.23±42.22 | 300.37±75.09 |
| Choroidal volume ($mm^3$): | | | | |
| outerT | 1.27±0.26 | 1.37±0.25 | 1.37±0.18 | 1.58±0.36 |
| innerT | 0.39±0.08 | 0.42±0.07 | 0.42±0.05 | 0.48±0.11 |
| central macular | 0.20±0.04 | 0.21±0.03 | 0.21±0.03 | 0.24±0.06 |
| innerN | 0.39±0.10 | 0.41±0.08 | 0.41±0.07 | 0.46±0.12 |
| outerN | 1.16±0.33 | 1.26±0.29 | 1.25±0.32 | 1.42±0.43 |
| outerS | 1.36±0.29 | 1.45±0.22 | 1.46±0.22 | 1.68±0.35 |
| innerS | 0.41±0.08 | 0.42±0.06 | 0.44±0.05 | 0.49±0.11 |
| innerI | 0.39±0.09 | 0.42±0.08 | 0.41±0.06 | 0.47±0.12 |
| outerI | 1.28±0.31 | 1.33±0.26 | 1.38±0.26 | 1.54±0.41 |
| total | 6.84±1.45 | 7.29±1.21 | 7.34±1.07 | 8.34±1.97 |
| Other choroidal parameters: | | | | |
| CVI | 0.59±0.06 | 0.58±0.05 | 0.60±0.04 | 0.63±0.05 |
| LA ($mm^2$) | 1.30±0.37 | 1.42±0.34 | 1.50±0.34 | 1.53±0.40 |
| SA ($mm^2$) | 0.90±0.21 | 1.03±0.22 | 1.01±0.22 | 0.87±0.19 |
| TCA ($mm^2$) | 2.19±0.51 | 2.45±0.50 | 2.51±0.51 | 2.40±0.53 |

Data presented as means ± SD

n = 216 eyes (76 eyes in the control group, 13 eyes with subretinal fluid, 89 eyes with cystoid macular oedema, and 38 eyes with diffuse macular oedema)

T = temporal; I = inferior; N = nasal; S = superior; SFCT = subfoveal choroidal thickness; CVI = choroidal vascularity index; LA = luminal area; SA = stromal area; TCA = total choroidal area; conventional ETDRS grid with nine subfields: central macular subfield (central field within a 500 μm radius), four inner subfields (within a 500-1500 μm radius), and four outer subfields (within a 1500-3000 μm radius)

Table S2. Univariate mixed-effect models (without covariates) comparing the study groups identified based on the presence of DME and type thereof (cystoid/diffuse/subretinal fluid vs. controls).

| Characteristic | Cystoid vs. controls (baseline) | | | Diffuse vs. controls (baseline) | | | Subretinal fluid vs. controls (baseline) | | |
|---|---|---|---|---|---|---|---|---|---|
| | **β** | **SE** | **p** | **β** | **SE** | **p** | **β** | **SE** | **p** |
| Choroidal thickness (μm): | | | | | | | | | |
| outerT | **-56.93** | **9.68** | **<.001** | **-41.87** | **11.27** | **<.001** | **-35.10** | **16.09** | **.030** |
| innerT | **-50.85** | **10.25** | **<.001** | **-42.29** | **12.13** | **.001** | **-36.15** | **17.52** | **.040** |
| central macular | **-46.08** | **10.63** | **<.001** | **-40.27** | **12.44** | **.001** | **-39.06** | **17.84** | **.030** |
| innerN | **-45.68** | **11.46** | **<.001** | **-42.26** | **13.41** | **.002** | **-39.75** | **19.23** | **.040** |
| outerN | **-46.80** | **12.39** | **<.001** | **-37.78** | **14.28** | **.009** | -23.46 | 20.19 | .246 |
| outerS | **-57.49** | **10.17** | **<.001** | **-46.62** | **11.78** | **<.001** | **-36.86** | **16.83** | **.029** |
| innerS | **-49.60** | **11.12** | **<.001** | **-41.89** | **11.88** | **.001** | -27.86 | 17.07 | .104 |
| innerI | **-46.89** | **11.09** | **<.001** | **-34.41** | **12.83** | **.008** | **-41.83** | **18.21** | **.023** |
| outerI | **-47.85** | **11.65** | **<.001** | **-36.59** | **13.31** | **.007** | -27.17 | 18.73 | .148 |
| SFCT | **-44.21** | **11.17** | **<.001** | **-34.88** | **13.31** | **.009** | -33.85 | 19.33 | .081 |
| Choroidal volume ($mm^3$): | | | | | | | | | |
| outerT | **-0.30** | **0.05** | **<.001** | **-0.22** | **0.06** | **<.001** | **-0.18** | **0.09** | **.032** |
| innerT | **-0.08** | **0.02** | **<.001** | **-0.06** | **0.02** | **.002** | -0.05 | 0.03 | .052 |
| central macular | **-0.04** | **0.01** | **<.001** | **-0.03** | **0.01** | **.001** | **-0.03** | **0.01** | **.032** |
| innerN | **-0.07** | **0.02** | **<.001** | **-0.08** | **0.02** | **<.001** | **-0.06** | **0.03** | **.042** |
| outerN | **-0.26** | **0.06** | **<.001** | **-0.21** | **0.07** | **.006** | -0.12 | 0.10 | .256 |
| outerS | **-0.31** | **0.05** | **<.001** | **-0.26** | **0.06** | **<.001** | **-0.20** | **0.09** | **.021** |
| innerS | **-0.08** | **0.02** | **<.001** | **-0.07** | **0.02** | **.001** | -0.04 | 0.03 | .110 |
| innerI | **-0.07** | **0.02** | **<.001** | **-0.05** | **0.02** | **.010** | **-0.07** | **0.03** | **.023** |
| outerI | **-0.25** | **0.06** | **<.001** | **-0.20** | **0.07** | **.005** | -0.17 | 0.09 | .087 |
| total | **-1.45** | **0.29** | **<.001** | **-1.15** | **0.32** | **<.001** | **-0.93** | **0.45** | **.039** |
| Other choroidal parameters: | | | | | | | | | |
| CVI | **-0.04** | **0.01** | **<.001** | **-0.05** | **0.01** | **<.001** | **-0.04** | **0.02** | **.015** |
| LA ($mm^2$) | **-0.20** | **0.07** | **.004** | -0.11 | 0.08 | .144 | 0.05 | 0.11 | .679 |
| SA ($mm^2$) | 0.03 | 0.04 | .455 | **0.14** | **0.04** | **.002** | **0.19** | **0.07** | **.004** |
| TCA ($mm^2$) | -0.17 | 0.09 | .070 | 0.03 | 0.11 | .815 | 0.24 | 0.16 | .135 |

β – coefficient from the regression model, SE – standard error, $p<0.05$ highlighted with bold
n = 216 eyes (76 eyes in the control group, 13 eyes with subretinal fluid, 89 eyes with cystoid macular oedema, and 38 eyes with diffuse macular oedema)
Each row represents one model with a factorial response variable (diffuse, cystoid, subretinal fluid), with the control group as a baseline

T = temporal; I = inferior; N = nasal; S = superior; SFCT = subfoveal choroidal thickness; CVI = choroidal vascularity index; LA = luminal area; SA = stromal area; TCA = total choroidal area; conventional ETDRS grid with nine subfields: central macular subfield (central field within a 500 μm radius), four inner subfields (within a 500-1500 μm radius), and four outer subfields (within a 1500-3000 μm radius)

Table S3. Univariate mixed-effect models (without covariates) comparing the study groups identified based on the type of DME (cystoid vs. subretinal fluid, diffuse vs. subretinal fluid).

| Characteristic | Cystoid vs. subretinal fluid (baseline) | | | Diffuse vs. subretinal fluid (baseline) | | |
|---|---|---|---|---|---|---|
| | β | SE | p | β | SE | p |
| Choroidal thickness (μm): | | | | | | |
| outerT | -21.76 | 12.49 | .084 | -6.63 | 12.75 | .604 |
| innerT | -14.77 | 14.52 | .311 | -5.32 | 15.11 | .725 |
| central macular | -7.89 | 14.80 | .595 | -1.15 | 15.35 | .940 |
| innerN | -5.50 | 16.51 | .740 | -0.75 | 17.06 | .965 |
| outerN | -21.35 | 17.06 | .213 | -10.74 | 17.45 | .539 |
| outerS | -20.18 | 14.08 | .154 | -8.75 | 14.39 | .545 |
| innerS | -20.71 | 13.99 | .141 | -12.16 | 14.45 | .402 |
| innerI | -6.06 | 15.14 | .690 | 7.04 | 15.62 | .653 |
| outerI | -21.16 | 15.66 | .179 | -9.96 | 16.02 | .536 |
| SFCT | -12.77 | 16.49 | .440 | -2.09 | 17.32 | .904 |
| | | | | | | |
| Choroidal volume ($mm^3$): | | | | | | |
| outerT | -0.12 | 0.07 | .078 | -0.04 | 0.07 | .583 |
| innerT | -0.03 | 0.02 | .234 | -0.01 | 0.02 | .788 |
| central macular | -0.01 | 0.01 | .583 | -0.002 | 0.01 | .850 |
| innerN | -0.008 | 0.03 | .777 | -0.01 | 0.03 | .615 |
| outerN | -0.13 | 0.09 | .127 | -0.08 | 0.09 | .388 |
| outerS | -0.11 | 0.07 | .144 | -0.05 | 0.08 | .526 |
| innerS | -0.04 | 0.02 | .111 | -0.02 | 0.02 | .380 |
| innerI | -0.01 | 0.02 | .668 | 0.01 | 0.02 | .639 |
| outerI | -0.09 | 0.08 | .261 | -0.03 | 0.08 | .702 |
| total | -0.51 | 0.35 | .153 | -0.19 | 0.35 | .594 |
| | | | | | | |
| Other choroidal parameters: | | | | | | |
| CVI | 0.003 | 0.02 | .863 | -0.005 | 0.02 | .787 |
| LA ($mm^2$) | **-0.24** | **0.10** | **.020** | -0.15 | 0.10 | .148 |
| SA ($mm^2$) | **-0.16** | **0.07** | **.014** | -0.05 | 0.07 | .465 |
| TCA ($mm^2$) | **-0.41** | **0.18** | **.007** | -0.21 | 0.15 | .172 |

β – coefficient from the regression model, SE – standard error, $p<0.05$ highlighted with bold
n = 140 eyes (13 eyes with subretinal fluid, 89 eyes with cystoid macular oedema, and 38 eyes with diffuse macular oedema)
Each row represents one model with a factorial response variable (diffuse, cystoid, subretinal fluid), with subretinal fluid as a baseline

T = temporal; I = inferior; N = nasal; S = superior; SFCT = subfoveal choroidal thickness; CVI = choroidal vascularity index; LA = luminal area; SA = stromal area; TCA = total choroidal area; conventional ETDRS grid with nine subfields: central macular subfield (central field within a 500 μm radius), four inner subfields (within a 500-1500 μm radius), and four outer subfields (within a 1500-3000 μm radius)

Table S4. Univariate mixed-effect models (with covariates) comparing the study groups identified based on the type of DME (cystoid vs. subretinal fluid, diffuse vs. subretinal fluid).

| **Characteristic** | **Cystoid vs. subretinal fluid (baseline)** | | | **Diffuse vs. subretinal fluid (baseline)** | | |
|---|---|---|---|---|---|---|
| | **β** | **SE** | **p** | **β** | **SE** | **p** |
| Choroidal thickness (μm): | | | | | | |
| outerT | -15.46 | 11.87 | .195 | -3.11 | 12.28 | .801 |
| innerT | -8.11 | 14.02 | .564 | 0.07 | 14.71 | .996 |
| central macular | -1.39 | 14.38 | .923 | 3.90 | 15.05 | .795 |
| innerN | 0.42 | 15.99 | .979 | 3.35 | 16.66 | .841 |
| outerN | -14.22 | 16.65 | .394 | -5.08 | 17.15 | .767 |
| outerS | -12.60 | 13.58 | .355 | -2.73 | 14.01 | .846 |
| innerS | -13.44 | 13.52 | .322 | -6.68 | 14.09 | .636 |
| innerI | 1.15 | 14.60 | .937 | 12.79 | 15.23 | .403 |
| outerI | -12.71 | 14.78 | .391 | -2.65 | 15.34 | .863 |
| SFCT | -5.88 | 15.91 | .712 | 3.49 | 16.85 | .836 |
| Choroidal volume ($mm^3$): | | | | | | |
| outerT | -0.08 | 0.06 | .184 | -0.02 | 0.07 | .778 |
| innerT | -0.02 | 0.02 | .462 | 0.001 | 0.02 | .959 |
| central macular | -0.001 | 0.01 | .944 | 0.002 | 0.01 | .851 |
| innerN | 0.004 | 0.02 | .877 | -0.003 | 0.03 | .915 |
| outerN | -0.09 | 0.08 | .248 | -0.05 | 0.09 | .579 |
| outerS | -0.07 | 0.07 | .334 | -0.02 | 0.07 | .822 |
| innerS | -0.02 | 0.02 | .284 | -0.01 | 0.02 | .653 |
| innerI | 0.001 | 0.02 | .985 | 0.02 | 0.02 | .414 |
| outerI | -0.05 | 0.08 | .499 | 0.002 | 0.08 | .983 |
| total | -0.34 | 0.34 | .322 | -0.06 | 0.35 | .859 |
| Other choroidal parameters: | | | | | | |
| CVI | 0.01 | 0.02 | .502 | 0.002 | 0.02 | .920 |
| LA ($mm^2$) | **-0.20** | **0.01** | **.047** | -0.12 | 0.10 | .228 |
| SA ($mm^2$) | **-0.17** | **0.07** | **.011** | -0.07 | 0.07 | .352 |
| TCA ($mm^2$) | **-0.37** | **0.15** | **.011** | -0.20 | 0.15 | .192 |

β – coefficient from regression model, SE – standard error, p<0.05 highlighted with bold
n = 140 eyes (13 eyes with subretinal fluid, 89 eyes with cystoid macular oedema, and 38 eyes with diffuse macular oedema)
Each row represents one model with factorial response variable (diffuse, cystoid, subretinal fluid), with subretinal fluid as a baseline
Covariates included in each model: age in years, sex (male/female), DR (NPDR/PDR), PRP (no/yes)

T = temporal; I = inferior; N = nasal; S = superior; SFCT = subfoveal choroidal thickness; CVI = choroidal vascularity index; LA = luminal area; SA = stromal area; TCA = total choroidal area; conventional ETDRS grid with nine subfields: central macular subfield (central field within a 500 µm radius), four inner subfields (within a 500-1500 µm radius), and four outer subfields (within a 1500-3000 µm radius)

Table S5. Univariate mixed-effect models (with covariates and without) comparing the study groups identified based on the type of DME (cystoid vs. diffuse).

| **Characteristic** | **Univariate models** | | | **Univariate models (sex, age, DR severity, PRP as covariates)** | | |
|---|---|---|---|---|---|---|
| | **β** | **SE** | **p** | **β** | **SE** | **p** |
| Choroidal thickness (μm): | | | | | | |
| outerT | **16.46** | **8.04** | **.043** | 13.52 | 7.62 | .079 |
| innerT | 10.25 | 9.64 | .290 | 9.09 | 9.14 | .322 |
| central macular | 7.07 | 9.70 | .468 | 5.75 | 9.23 | .534 |
| innerN | 5.95 | 10.82 | .584 | 4.43 | 10.33 | .669 |
| outerN | 11.44 | 10.71 | .288 | 10.67 | 10.39 | .306 |
| outerS | 10.70 | 9.04 | .239 | 9.41 | 8.68 | .281 |
| innerS | 8.34 | 9.10 | .361 | 6.73 | 8.64 | .437 |
| innerI | 13.83 | 9.89 | .165 | 12.46 | 9.49 | .191 |
| outerI | 11.08 | 9.51 | .246 | 10.92 | 9.11 | .233 |
| SFCT | 10.92 | 10.86 | .317 | 9.77 | 10.26 | .343 |
| Choroidal volume ($mm^3$): | | | | | | |
| outerT | **0.09** | **0.04** | **.042** | 0.07 | 0.04 | .079 |
| innerT | 0.02 | 0.02 | .155 | 0.02 | 0.01 | .209 |
| central macular | 0.004 | 0.008 | .569 | 0.003 | 0.007 | .641 |
| innerN | -0.003 | 0.02 | .862 | -0.002 | 0.02 | .887 |
| outerN | 0.06 | 0.05 | .259 | 0.06 | 0.05 | .269 |
| outerS | 0.06 | 0.05 | .234 | 0.05 | 0.05 | .273 |
| innerS | 0.01 | 0.01 | .303 | 0.01 | 0.01 | .343 |
| innerI | 0.02 | 0.02 | .149 | 0.02 | 0.02 | .179 |
| outerI | 0.06 | 0.05 | .256 | 0.06 | 0.05 | .239 |
| total | 0.33 | 0.23 | .151 | 0.31 | 0.22 | .176 |
| Other choroidal parameters: | | | | | | |
| CVI | -0.007 | 0.01 | .560 | -0.008 | 0.01 | .456 |
| LA ($mm^2$) | 0.11 | 0.06 | .069 | 0.10 | 0.06 | .099 |
| **SA ($mm^2$)** | **0.12** | **0.04** | **.005** | **0.12** | **0.04** | **.008** |
| **TCA ($mm^2$)** | **0.23** | **0.09** | **.013** | **0.21** | **0.09** | **.021** |

β – coefficient from the regression model, SE – standard error, $p<0.05$ highlighted with bold
n = 126 eyes (89 eyes with cystoid macular oedema, and 38 eyes with diffuse macular oedema)
Covariates included in each model: age in years, sex (male/female), DR (NPDR/PDR), PRP (no/yes)

T = temporal; I = inferior; N = nasal; S = superior; SFCT = subfoveal choroidal thickness; CVI = choroidal vascularity index; LA = luminal area; SA = stromal area; TCA = total choroidal area; conventional ETDRS grid with nine subfields: central macular subfield (central field within a 500 μm radius), four inner subfields (within a 500-1500 μm radius), and four outer subfields (within a 1500-3000 μm radius)
